# Autobiographical Therapeutic Performance as a Means of Improving Executive Functioning in Traumatized Adults

**DOI:** 10.3389/fpsyg.2021.599914

**Published:** 2021-02-12

**Authors:** Paula Ray, Susana Pendzik

**Affiliations:** ^1^Private Psychology Practice, Lincoln, NE, United States; ^2^Graduate Program in Drama Therapy, Tel Hai Academic College, Upper Galilee, Israel

**Keywords:** autobiographical therapeutic performance, executive functioning, traumatized adults, drama therapy, community theater, job readiness, family support

## Abstract

This article describes the pilot project *Shadows* & *Light Within: Untold Stories*—a two-phase, multi-partner community-based project that explores the hypothesis that Autobiographical Therapeutic Performance can help traumatized individuals to improve executive functioning. A group of 10 individuals ranging in age from 32 to 69, with lived experiences at the intersection of trauma, mental health, and the court system, were paired with theater mentor-coaches for a 10-month creative group process, in which they shaped their stories into autobiographical performance pieces, through movement, improvisation, story-telling, and self-discovery. In the second phase of the project, their stories were merged into a theater production, weaving movement, song, and voice, and performed by an ensemble of experienced actors from the community. Pre- and post-interviews and self-report standardized measures of executive functioning were used to assist in establishing criteria and direction for future research. The results suggest that the individuals involved in this pilot may have improved executive functioning and acquired more ability to engage in human service programs designed to increase job readiness and enhance adaptive living skills.

## Introduction

*Shadows* & *Light Within: Untold Stories* explores if and how the process of developing, performing, and witnessing autobiographical narratives using drama therapy interventions can lead to improved executive functioning for individuals with trauma history, in an attempt to facilitate their engagement in human service programs designed to increase job readiness. The project was conceived as a model community partnership between a nonprofit organization providing advocacy and support for families, and a nonprofit theater company committed to community engagement and improving the quality of life through artistic means. The project involved adults with histories of personal and family mental illness and maladaptive coping that resulted in legal/court involvement and placement of their children into state custody. A group of 10 individuals, aged 32–69, were invited to develop an Autobiographical Therapeutic Performance (ATP) based on their lived experiences. During the second phase, their ATPs were merged into a staged production presented by community theater performers, incorporating movement and music, with the original participants as members of the larger and preselected audience.

Quite often, individuals who find themselves at the intersection of mental health and the court systems have experienced multiple traumas, growing up in chaotic and sometimes violent environments. According to Siegel (2003), toxic stress associated with trauma can alter brain development and functioning, particularly in the orbitofrontal region of the brain, which is vital in developing a cohesive narrative of one's life with an integrated storyline of one's past, present, and anticipated future. Brain research has established a relationship between neural networks involved in developing a cohesive autobiographical narrative and executive functioning (EF)—often referred to as a set of goal-directed, future oriented cognitive skills (Tsermentseli and Poland, 2016) that are essential for adaptive functioning. Executive functions comprise the higher-level influences over sensory input, emotional and cognitive internal states, and motor output, thereby providing dynamic integration of external and internal environments (Gazzaley and D'Esposito, 2007).

Distinctions have been made in the literature between “cold” and “hot” executive functioning, associated with different regions of the frontal lobes and with somewhat different aspects of EF. The “cold” EF is associated with dorsolateral prefrontal cortex functioning and is evoked under nonaffective, or relatively abstract situations (Zelano and Carlson, 2012). The “hot” EF is associated with the orbitofrontal and ventromedial regions, which overlap and are strongly related to the limbic regions, associated with emotional and social processing (Peterson and Welsh, 2014). Within the framework of the Polyvagal Theory (Porges, 2011), social processing involves the autonomic nervous system which mediates a pre-conscious process on a neurophysiological level involved in detection of a person as safe or dangerous, thus determining prosocial or defensive behavior. Recovery from trauma, whether natural or accomplished through psychotherapy, involves the organization and streamlining of fragmented memories (Foa et al., 2007), integrated with change in social processing at a neurophysiological level.

Autobiographical Therapeutic Performance (ATP) is a therapeutic intervention that involves developing theater pieces based on personal material and performing them in front of an audience (Pendzik et al., 2016). As a particular form of drama therapy called performance-based, in ATP the performative frame constitutes the therapeutic setting. Drama therapy in this modality “takes place within the special time and space of the creation of a performance which eventually has an audience and a post-performance review” (Snow, 2009, p. 117). ATP differs from Autobiographical Theater in its declared therapeutic intent, which requires some sort of therapeutic accompaniment (Pendzik, 2020). The approach is essentially based on three assumptions: (a) The act of “storytelling” aspects of our lives generates more coherent self-narratives, which may help to better integrate and cope with traumatic life events (White and Epston, 1990; McAdams, 2008). (b) Transforming these narratives into live performances challenges individuals to take concrete and embodied actions that may help to solidify constructive self-narratives (Emunah et al., 2014; Pendzik, 2016) and to consolidate the repaired experience as a long-term memory item (Yaniv, 2014). (c) Performing in front of an audience validates the alternative narratives created, giving them a public scope that intensifies their healing potential (Sajnani, 2012, 2016; Emunah, 2015, 2016).

These three factors (storytelling, embodiment, and performance) have been recognized as having therapeutic implications. Storytelling seems to be an inherent human resource for integrating life experience and healing from trauma (Haven, 2007; Lahad, 2019). Embodiment is becoming a topic of interest in cognitive science with a key assumption that the mental processes we associate with cognition are fundamentally linked to bodily processes, which inform EF (Koziol et al., 2012)—an idea that has been explored in theater (Olenina et al., 2019). Therapeutic Performance has been found to help people to theatrically “work through” lived experiences (Emunah, 2020), facilitating the integration of personal experiences in the social sphere. The present study explores the idea that through the embodied performance of one's personal narrative, as done in Autobiographical Therapeutic Performances, a dynamic point of neural integration can be strengthened (Yaniv, 2014), leading to improved EF, which in turn could get people better prepared to engage in human services designed to facilitate their social integration and job readiness.

A further progression that this study seeks to assess concerns the therapeutic implications of elaborating a full professional piece based on the personal material provided by the “source”—the person whose autobiographical material is being performed (Hodermarska et al., 2016). In the current project, this phase involved a discrete participation of the sources in the rehearsals, in order to confirm their resonance with the artistic creation in-the-making, and finally, becoming an audience of their story in a wider public performance.

## Methodology

### Phase 1

Ten individuals (*n* = 10) 9 women and 1 male, ranging in age from 32 to 69, 4 Hispanic, 1 African American, and 5 Caucasian, were recruited primarily from within the existing clientele of a nonprofit organization that provides support to families experiencing mental, emotional, and behavioral health challenges. The project design and the process of developing ATPs were presented during a regularly scheduled family support group. Interested people were informed of the criteria for participation that included commitment to attend the group twice per month for 10 months and to meet and maintain regular contact with an assigned coach. An informed consent form, including steps to address grievances and/or to drop out of the project, was reviewed and signed by each person who agreed to participate.

Each participant was asked to complete a standardized self-report measure of executive functioning in daily life and assigned an individual coach to assist in the shaping of their personal narrative into an ATP to be shared within the group at the end of the 10-month period. The coaches were recruited from within the partnering community theater company and consisted of individuals with experience in acting, directing, playwriting, and theater production. Coaches were provided with ongoing training in the project modality and regular supervision sessions. Following the recruitment phase, participants met bi-monthly as a group from May 2019 to February 2020 and engaged in drama therapy interventions facilitated by the project director and theater coaches. Continued guidance and support were provided outside of the group by the coaches, to assist in shaping the narratives into ATPs to be performed within the small group setting.

### Phase 2

In phase 2, a narrative thematic analysis (Braun and Clarke, 2012) of the ATPs provided the source material that was merged into an ensemble script, using design principles that combined both realistic and symbolic modes of presentation in order to encourage audience resonance (Wood, 2018). To allow for objective viewing of their personal source material and subjective intrapersonal self-reflection, in phase 2, participants did not perform themselves but were engaged in the editing of the final ensemble script, to ensure the integrity of their stories. For this part, actors were auditioned from the local community theater and connected with the participants whose source material they were representing. This enabled a continuing dialogue between participants and the performance ensemble. The initial performance was postponed due to the Coronavirus pandemic and performed 3 months later for a closed audience comprised of participants, coaches, invited guests, and others involved in the project. Three additional performances were held for general audiences.

## Results

### Post-participation Interview and EF Self-Report

All participants attended a minimum of one performance. Post-evaluation interviews were completed, and self-report standardized measures of EF (BRIEF-A) were administered within the 2-week period following the production. One participant moved to a different location and was not available for follow-up. In the structured interview, participants were asked 6 questions (see Table 1), concerning their experience of sharing their ATPs in a small group, viewing a performance developed from their source material, as well as their reflections on the assigned coaches, and other comments they had on the experience.

**Table 1:** Post-evaluation interview questions.

➢ How was it for you to perform your autobiographical performance in the group?
➢ What was it like for you to work with the coach?
➢ How was it for you to view the ensemble performance?
➢ How do you view your involvement in the project?
➢ Have you had a change in perspective or behavior that you connect with your involvement with the project?
➢ Other thoughts? Comments?

### Qualitative Summary of Post-participation Structured Interview Responses

A qualitative feminist paradigm informed our study (Doucet and Mauthner, 2007). In our approach to participants' responses, particular attention was paid to issues related to power, relational mutuality, race, and social awareness.

Many participants reported starting the process feeling anxious, nervous, overly vulnerable, exposed, and scared of how people would judge them; some even said they did not want to do it at first. With encouragement from their assigned coaches and group support, these feelings were mostly dispelled as the process advanced, and replaced by a sense of being proud of themselves, feeling open and more accepted:

- *Awkward, out of my comfort zone, but (….) I'm proud of myself for putting myself out there—never done that before and I'm proud of myself*.
- *Nervous, didn't want to do it. Didn't want to share—I was nervous how people would look at me. I broke some heavy themes in my life—I expressed things I always wanted to express*.
- *First, I didn't get it. By the end I did. It was scary and good*.

The aid of the coach was extremely valuable to participants. Some expressed that the coach was instrumental in keeping them “on track” and encouraging them to take balanced emotional risks; others claimed that the coach helped them to organize the experience as a narrative and even to develop a skill for doing that. Many referred to the relational aspects of this pairing: issues of trust, vulnerability, self-esteem, and race emerged.

- *We developed a relationship—we're friends now. Very interesting to make life experiences into a script, a real skill and I evolved by doing it. Didn't know it could be expressed that way*.
- *Helped a lot. Kept me on track. She helped me feel vulnerable and be OK*.
- *Thought it wouldn't work—was worried she was a “uppity white girl.” She was the best coach ever. She brought me out of my shell, helped me trust what I didn't trust*.

Regarding the witnessing of the performance, common themes recurred throughout the participants' responses: A feeling of being deeply “moved” by it, combined with a sense of validation and being accompanied by others; an appreciated sense of distance from the actual experience; and many references to the aesthetic aspects of the performance:

- *I was so moved by the songs! Hearing the stories of others was validating for me*.
- *Gave me a distance from myself. I was connected to it but not triggered by it emotionally, like I have been. Lightened the charge of that situation. I was involved in the actors doing their parts and applauding the fact it was woven together*.
- *I thought the sounds, movement and transitions flowed together really well—told our stories in a real way. I felt it and watched without wanting to hide*.
- *Beautiful—it showed me we're human, and go through things, and be able to grow empathy for others. I felt less alone. It's like birds chirping—a group chirping together—sounds like music. Touching—movement and music. I heard and really felt it*.
- *Amazing, very cool, neat. No words to describe. I'm watching and felt exposed in a good way, for the first time, in a positive way. Like a unique feeling, finally, I closed that book. Resolved, without shame, crying, no secrets anymore*.

The overall response to the project revealed a general sense of acceptance, that something had been “resolved.” Participants report being clearer, lighter, and more at ease with their lived experiences:

- *Fascinating. (The) trauma felt more resolved. It's settled. Much stronger connections. As a parent, I have less worry and feel free from some weight*.
- *More acceptance—acceptance on so many different layers…*
- *Resolved—my self-perspective is clearer. Like now we can put the band aid on the wound. I feel now I can move on with my life*.

In addition, many participants attributed to the process personal changes of perspective and behavior, particularly concerning relationships with others—such as developing empathy and understanding toward others, as well as a more reflective and relaxed attitude toward life.

- *Helped me prepare for my job now—I work with people and I am more aware of their feelings. I'm kinder to my mom now, I understand her more. I don't feel the need to cry anymore when I feel I'm not good enough for my boyfriend…*
- *I'm not using CPAP (continuous positive airway pressure) as much—not having as many incidents. Less disrupted sleep. Allowing myself to not work as much—read and relax more. More reflection on my connections with others. Less quantity, more quality*.
- *I took it home… (I) pay more attention to my parenting skills, my tone of voice, how much time I'm on the phone. I understand so much more. I wouldn't have thought how I set the tone (at home). I reflect more on myself, I calm down, more open-minded*.

All participants reported positive results, saying that the experience brought them closer to other people; one participant suggested that it would be good that people who work in the system would see the performance, and another lamented that the pilot ended. All of them (without exception) said they would recommend the project to others.

### Standardized Self-Report of EF in Daily Life (BRIEF-A)

Pre- and post-administration of the Behavior Rating Inventory of Executive Function®-Adult Version (BRIEF®-A) was obtained from 9 of the 10 project participants. The BRIEF-A is a standardized rating scale developed to provide a window into everyday behaviors associated with specific domains of EF in adults ages 18–90 years (Roth et al., 2005). The BRIEF-A Self-Report consists of 75 items in 9 nonoverlapping subscales that comprise 2 summary index scales, the Behavioral Regulation Index (BRI) and the Metacognition Index (MI), and a scale reflecting overall functioning Global Executive Composite (GEC). The Behavioral Regulation Index (BRI) captures the ability to maintain appropriate regulatory control of one's own behavior and emotional responses. Consistent with the concept of dual “hot and cold” aspects of EF, the authors suggest that appropriate behavioral regulation is likely to be a precursor to appropriate metacognitive problem solving. It enables the metacognitive processes to successfully guide active and systematic problem solving, as well as more generally supporting appropriate self-regulation. The Metacognition Index (MI) reflects the individual's ability to initiate activity and generate problem-solving ideas, to sustain working memory, to plan and organize problem-solving approaches, to monitor success and failure in problem solving, and to organize one's materials and environment. The Global Executive Composite (GEC) scale reflects overall functioning. T scores (M = 50, SD = 10) are used to interpret the individual's level of executive functioning. Lower scores indicate less report of problems. T scores at or above 65 are considered elevated to be within clinically significant range.

Table 2 displays the pre- and post-process scores on the 3 major indices for 9 of the 10 participants. Pre-process measures were obtained in March and April 2019, and post-process measures in July 2020.

**Table 2:** Pre and post T score comparison.

	**Participants**									
	**Evaluation period**	**1**	**2**	**3**	**4**	**5**	**6**	**7**	**8**	**9**
BRI	Pre-process	80	60	72	51	50	57	51	53	90
	Post-process	62	51	63	58	55	52	54	59	63
	Amount of shift (pre–post)	−18	−9	−9	+7	+5	−5	+3	+6	−27
MI	Pre-process	81	60	85	55	59	71	48	54	92
	Post-process	65	53	61	54	56	64	53	48	70
	Amount of shift (pre–post)	−16	−7	−24	−1	−3	−7	+5	−6	−22
GEC	Pre-process	83	61	82	54	56	66	49	54	94
	Post-process	65	52	63	57	56	60	54	53	68
	Amount of shift (pre–post)	−18	−9	−19	+3	0	−6	+5	−1	−26

As shown in Table 2, 3 of 9 participants obtained pre-process scores on the BRI scale within the clinically significant range compared to post-process scores within normal range, indicating self-report of increased capacity for appropriate modulation of emotional response, inhibition of thoughts and actions, increased mental flexibility in problem-solving, and monitoring of one's actions. Five participants obtained pre- and post-process scores within the normal range with some variation in subscale reporting, and 1 participant obtained a pre-process score of 60 and post-process score of 51, indicating report of decreased difficulty. Results on the MI scale show pre-process scores within the clinically significant range for 4 individuals, with 2 of the 4 obtaining post-process scores within normal range, and 2 remaining within the clinical range but with decreases of 16 and 20 points, indicating self-report of improved ability to generate and initiate problem-solving activity and sustain working memory, organize their environment, and monitor success and failure in problem solving. One participant obtained an MI pre-process score of 60 and a post-process score of 53, indicating report of improved functioning; 4 participants obtained pre- and post-scores within the normal range with some variation in subscale reporting.

## Discussion

*Shadows* & *Light Within: Untold Stories* explored the application of a drama therapy performative frame (Snow, 2009) to help individuals improve EF, thereby acquiring a fuller perspective on themselves and the world, and facilitating their engagement in human service programs designed to increase adaptive functioning and job readiness. The results of this pilot project suggest that the guided therapeutic process of transforming lived experience into embodied narratives during ATPs, and later, viewing an aesthetically enhanced version of this material, may have increased participants' critical thinking skills and creativity in problem solving—thereby gaining more control of their ability to guide and manage emotional, cognitive, and behavioral functions.

The changes noted in the pre- and post-self-reporting measures of EF in daily life combined with reports of the subjective experience of several participants appear to support this idea. One participant obtained a decrease from the pre-process T-score on the Metacognitive Index (MI) within the clinically significant range (T = 71) to the post-process score within the normal range (T = 64), reporting sleep improvement with less need for use of CPAP and that “the charge of it (trauma) lightened and felt resolved.” Another participant obtained a decrease on the Behavioral Regulation Index (BRI) in the pre-process T-score within the clinically significant range (T = 80) to the post-process score to within the normal range (T = 62) combined with drop in pre-process MI within the clinically significant range (T = 81) to the post-process score within the normal range (T = 65). Subjectively, this participant described the experience in terms of “validation,” “universal,” and clearer “self-perception,” reporting improved self-reflection, calmness, and enhanced parenting skills and interpersonal relations. A third participant displayed the same pattern of score changes on both the BRI and MI indices from pre-process scores within a clinically significant range to post-process scores within the normal range (BRI pre T of 72 to post T of 63, MI pre T of 85 to post T of 61), combined with interview comments of feeling “resolved” and “finally closing that book, without shame, crying…no secrets anymore.” One participant described the project as helping “to prepare for my job now.”

In trying to understand these tendencies, we noted that similar versions of the first phase of this project have been successfully applied with diverse populations: ATPs have produced valuable results with people diagnosed with psychiatric conditions (Emunah, 2015, 2020), survivors of childhood trauma and abuse (Woodland, 2009; Emunah et al., 2014; Gopalakrishna and Rao, 2017), prison inmates (Daccache, 2016), older people (Harel, 2016), and people suffering of collective trauma (Volkas, 2016), among others. However, whereas these interventions typically conclude in a culminating performance by the person presenting their own lived experience, including a subsequent integration phase, the current project added the transformation of the material into an aesthetically devised performance, with discrete involvement of the sources, in order to ensure the integrity of the piece. This distinction is important because sharing their own ATP involved a social/emotional component, whereas viewing the large performance relied more heavily on each participant's subjective intrapersonal reflection of being an audience to someone else's retelling of their own source material, allowing for increased objective witnessing and self-reflection.

With few exceptions, this particular procedure differs from more “classical” models of ATP. In a somewhat comparable intervention, military veterans were asked to write a monolog of their own trauma based on Shakespeare's monologs and then watched it being performed by a fellow veteran (Ali et al., 2018). The researchers concluded that this intervention encouraged “self-forgiveness” “because it becomes possible to forgive another for actions that you might blame yourself for” (p. 158). Likewise, Hodermarska et al. (2016) experimented with the idea of the “play as client” by separating four functions: source, playwright, director, and performer. In that study, the source provided personal material to a playwright, who created a script and passed it on to a director, who in turn directed a performer. The source never saw the script nor discussed it with the director. Witnessing the play as an audience for the first time, the source reported experiencing a profound sense of catharsis and revelation: “I was at that moment, in the deepest existential sense, not alone” (p. 263). This response coincides with those of the participants in the current project: While the first part of the process was described as a valuable building block that included creating trust, allowing themselves to be vulnerable, overcoming fears of being judged, and developing relational as well as narrative skills, the witnessing phase seemed to elicit a feeling of belonging and of collective awe that border a sense of *communitas*. As Turner (1982) suggested, this is a state of great intensity that uplifts participants from the constraints of ordinary life, allowing them to meet each other in a direct and intimate encounter. It is here where actual transformation takes place.

It is significant to note that participants' responses to the question of witnessing the performance (Table 1, Q3) rendered the most abundant and rich answers—including aesthetics remarks, references to the universality of human experiences, and a sense of resolution or transformation of the trauma. The place of the audience in ATP is a crucial one (Sajnani, 2012, 2016; Emunah, 2016, 2020). Volkas (2016) highlights its function as a “witness” that “provides a reparative holding and mirroring experience” for participants (p. 125), “paint(ing) their individual struggles with a collective brush” (p. 126). Unlike the “classical” ATP structure, in which performers undergo a “rite of passage” as themselves, in the second phase of this project participants also witnessed their own stories being transformed through the alchemy of aesthetics into artistically crafted pieces, thus also functioning as reparative witnesses of their own lived experience. It appears that this level added a further social dimension to the elaboration of the trauma. Perhaps in this sense, the involvement of the community theater signaled participants that society at large is now acknowledging and taking some responsibility for their traumatic lived experience.

A similar occurrence of witnessing one's lived experiences being performed by skilled actors is found in Playback Theater. There, members of the audience are invited to go on stage and tell personal stories to a conductor, who then hands these over to an ensemble of trained performers (Salas, 2009). As Playback Theater is an improvisational form, the whole process takes place on the spot, and the teller is usually not involved in the performance, except for being the provider of the story. Although Playback Theater aims to create empathic and healing renderings of the story, there are always risks of appropriation or misunderstandings, which in a performance mode, may leave tellers with a sense of being misrepresented (Rowe, 2007), thereby problematizing their ability to self-reflect. This project applied a different methodology: The piece was fashioned through a complex and gradual process that included elaborating the lived experience first in dyads (with the coach), then in the small group, and finally, at the level of the community (larger audience). Throughout all these phases, there was an interactive process of de/constructing and re/constructing the lived experience in a relational dialogue, which allowed participants to “take in” the reframed narratives and elaborate their experience by inhabiting the double role of participant and observer that characterizes aesthetic distance (Landy, 1996). Perhaps through these combined interactions, “hot and cold” aspects of EF became actively implicated, in a way that enabled metacognitive processes to develop.

Jacques (2016) poses that *alterity* is a sine qua non condition of theatrical phenomenology, which requires the eye of the “other” in order to create completion. He articulates five levels of witnessing, which may account for some of the therapeutic attributes of ATPs. These include the “internal witness” (performers), “engaged witness” (group members), “active witness” (director), “observing witness” (external audience), and “silent witness” (larger historic/cultural context). In the *Shadows & Light Within* project, participants got to consciously play at least four of these levels (the fifth entails an unconscious, collective process): They were internal witnesses of their own performances and engaged witnesses in the group; they played active witnesses during the rehearsal of the large performance, and observing witnesses of their own stories in the larger audiences. Inhabiting all these levels of reflection may have contributed to improve the participants' EF at the end of the project, as evidenced by the changes recorded on the scores of standardized self-report measures.

Emunah (2016) points out that audiences are also emotionally touched by ATPs: “Audience members who know the performer intimately may be particularly stirred, with feelings of empathy, care, love, and awe for the person on stage” (p. 47). In her view, the artistry of the piece may help to hold the unnecessary concern that the performer's struggles may elicit in close audience members. In the same vein, Wood (2018) points out that “in order for the audience to connect emotionally with the piece, there needs to be balance between authenticity and skill” (p. 29). In a qualitative research that examines how witnessing performances based on lived experience can educate and heal families, Wood (2018) states that audiences reported resonance with the performance due to a balanced approach to realistic and symbolic modes of representation, claiming that participants expressed that the artistic approach offered a sense of safety and containment and that the aesthetic distance of the medium provided a layer of protection. All this holds true for the participants the *Shadows & Light Within* project, who, in the role of witnesses, were moved by the personal intensity of their lived experience, and at the same time, felt protected by the aesthetic mastery of the performance.

## Limitations

Autobiographical memory cannot be reduced to a single memory system or process. As McKinnon et al. (2007) assert, due to this complexity it cannot be presented in a controlled environment and any study of autobiographical memory must represent a compromise between experimental control and real-life relevance. Upon reflection of the data, it would have been interesting to administer the self-report of executive functioning in daily life (BRIEF-A) also at the end of phase one, in order to determine if the changes in the executive functioning improved more after the personal presentation. The use of a pre- and post-measure of job readiness and adaptive functioning, such as the Career Orientation Placement and Evaluation Survey (COPES), would have allowed for a more comprehensive analysis of the outcomes, as well as a follow-up in 3–6 months to assess if participants benefited from the intervention and comparison with a control group would have provided useful information. However, given the eruption of the Coronavirus pandemic, it is questionable whether the positive outcomes of the project would have translated into job findings by the participants. In addition, *Shadows & Light Within: Untold Stories* was a pilot study, a model community program design. Future projects would benefit from an underpinning research of combined qualitative and quantitative methods like the ones we just mentioned.

## Conclusions

The *Shadows* & *Light Within: Untold Stories* project explored the role of creating an ATP out of lived experience, and later witnessing a professional rendering of it—fashioned with some involvement by the participants. The assessment of the whole process was observed vis-à-vis the improvement of the participants' EF. Although the study was limited to 10 participants, its results suggest that the individuals involved in this pilot project who have lived experiences at the intersection of trauma, mental health, and court systems may have more ability to engage in the human service programs designed to enhance adaptive living skills in daily life. As the trauma tool-kit issued by the U.S Department of Health and Human Services, Administration for Children & Families (https://www.acf.hhs.gov/trauma-toolkit/executive-function) reports, improved executive functioning skills will be needed to promote engagement in these programs for individuals who have been impacted by toxic stress, trauma, and other adverse experiences. Hence supporting further research that back projects of this sort seems extremely advantageous for everyone involved.

## Data Availability Statement

The raw data supporting the conclusions of this article will be made available by the authors, without undue reservation.

## Ethics Statement

Ethical review and approval was not required for the study on human participants in accordance with the local legislation and institutional requirements. The patients/participants provided their written informed consent to participate in this study.

## Author Contributions

PR created and implemented the project design and contributed to the Introduction, Methodology, Results, Discussion, and Conclusions sections. SP articulated the paper and conceptualized the link between drama therapy theory and the results of the study, contributing to the analysis, and writing of the paper. All authors contributed to the article and approved the submitted version.

## Conflict of Interest

The authors declare that the research was conducted in the absence of any commercial or financial relationships that could be construed as a potential conflict of interest.
